# Hepatocellular carcinoma with ring calcification mimicking hydatid disease: a case report

**DOI:** 10.1186/s40792-020-00927-5

**Published:** 2020-07-13

**Authors:** Yutaro Matsunaga, Shunichi Ariizumi, Go Shibuya, Shuichiro Uemura, Takaaki Kato, Takehisa Yazawa, Shingo Yamashita, Akiko Omori, Ryota Higuchi, Yutaka Takahashi, Yoshihito Kotera, Hiroto Egawa, Masakazu Yamamoto

**Affiliations:** Department of Surgery, Institute of Gastroenterology, Tokyo Women’s Medical University, 8-1Kawada-cho, Shinjuku-ku, Tokyo, Japan

**Keywords:** HCC, Calcification, Ring calcification, Rim calcification, CT findings, Echinococcosis, Hydatid disease

## Abstract

**Background:**

Ring calcification in hepatocellular carcinoma is extremely rare. Untreated hepatocellular carcinoma occasionally includes calcified lesions. Here, we report a case of ring-calcified hepatocellular carcinoma.

**Case presentation:**

A 60-year-old man with a hepatic tumor was referred to Tokyo Women’s Medical University Hospital. He had a history of chronic hepatitis C. Computed tomography showed a liver tumor 20 mm in diameter in segment 6 of the Couinaud classification, with ring calcification. Based on this uncommon imaging presentation and the patient’s past exposure to the definitive hosts of *Echinococcus multilocularis*, he was preoperatively diagnosed with echinococcosis. Partial hepatectomy was performed as a radical treatment for echinococcosis. A final diagnosis of hepatocellular carcinoma was confirmed based on pathological findings. The patient was discharged uneventfully.

**Conclusion:**

The presentation of an extremely rare hepatocellular carcinoma with ring calcification may be disguised as hydatid disease.

## Background

Calcification of untreated hepatocellular carcinoma (HCC) has been reported in 3.3–25.0% of HCC cases [1–4]. Calcification of untreated HCC is less common than fibrolamellar carcinoma [4–6], metastatic liver tumor, or hemangioma [6, 7]. The patterns of calcification may characterize specific hepatic lesions [6, 8]. Multiple, ill-defined patterns are associated with metastatic liver tumors. A solitary, stellate, central-located pattern is associated with fibrolamellar carcinoma. The turtle back pattern is unique to schistosomiasis. A peripheral rim pattern, which resembles ring calcification, is often seen in echinococcosis [6, 8, 9].

## Case presentation

A 60-year-old man with asymptomatic chronic hepatitis C was referred for a hepatic tumor that was detected on screening abdominal ultrasonography. Laboratory data showed that serum levels of alpha-fetoprotein and protein induced by vitamin K absence-II were not elevated; indocyanine green had a 27% retention rate at 15 min, the Child-Pugh score was 5, and liver damage was “A.”

**Fig. 1:**
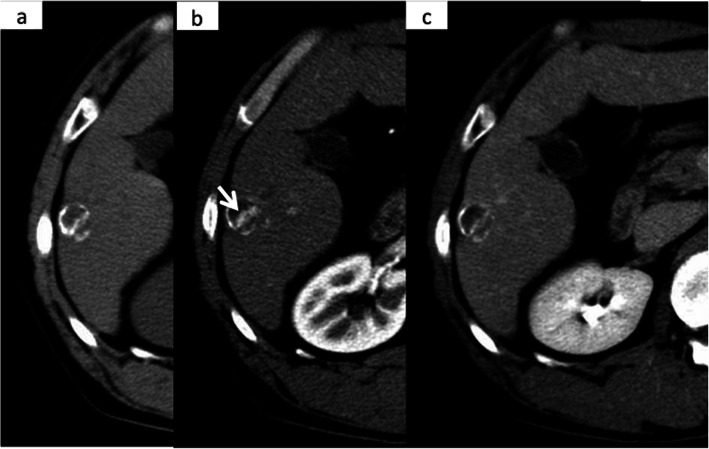
Dynamic CT. **a** A calcified tumor was seen in segment 6 of the liver in the plain CT. **b** CT in the arterial phase showing partial early enhancement of the tumor (indicated by white arrows). **c** CT in the delayed phase showing a washed-out pattern in the tumor

Computed tomography (CT) revealed a 20-mm well-defined tumor with calcification in segment 6 of the Couinaud classification of the liver. Calcifications were distributed, especially on the edge of the tumor. Ring calcification was observed in the tumor (Fig. 1a). Dynamic CT showed a typical HCC pattern, which was enhanced in the early phase and washed out in the delayed phase in the center of the tumor (Fig. 1b, c).

The environmental factor should be described since echinococcosis was suspected due to the presence of ring calcification in the liver. Although biochemical analysis of the echinococcus antibody was negative, the patient’s unusual past engagement in red fox hunting in the Hokkaido area led to an association with echinococcosis. Based on this unusual environment factor and the characteristic ring calcification, a preoperative diagnosis of echinococcosis was made. To treat, partial liver resection of segment 6 was performed.

**Fig. 2:**
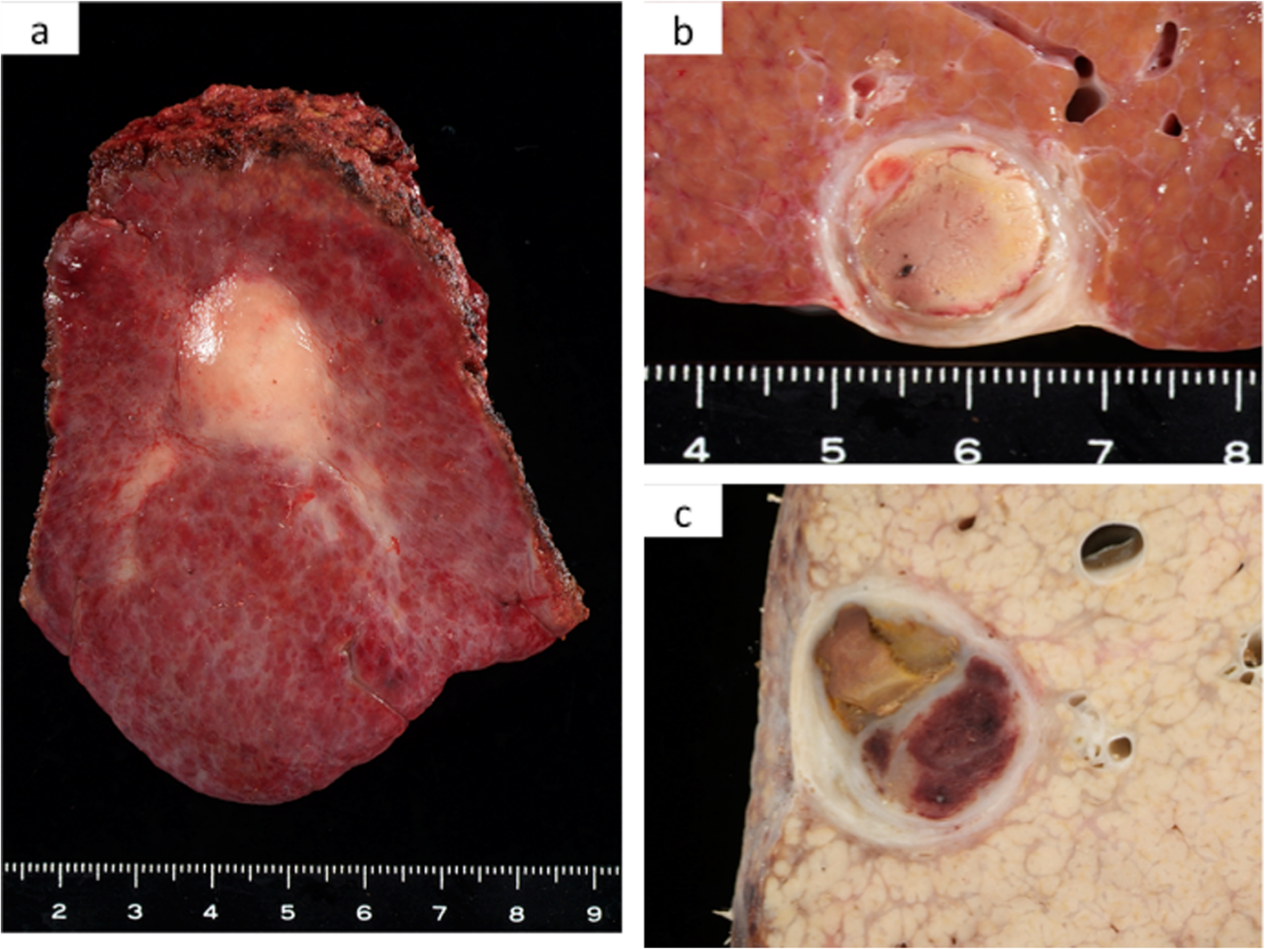
Macroscopic image of the tumor. **a** A solid tumor was seen on the surface of the liver. **b** A cross section of the tumor revealed the thick capsule. **c** Another cross section of the tumor after formalin-fixation showed hemorrhage and necrosis inside the tumor

**Fig. 3:**
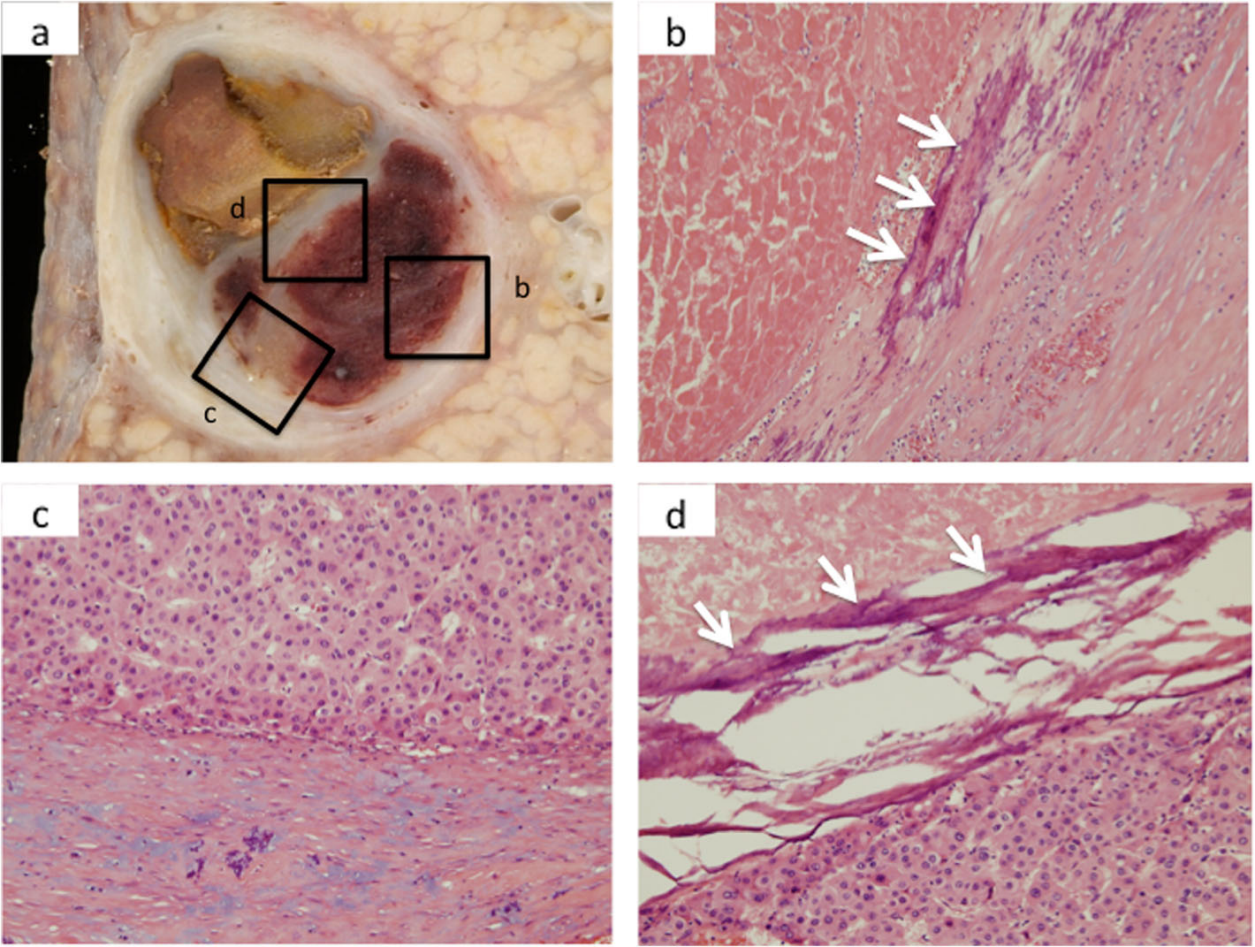
Microscopic findings of the tumor. **a** A cross-section of the tumor. Upper-case alphabets with windows correspond to microscopic images labeled with lower-case alphabets. **b** Image around the capsule showing hemorrhagic area along with rim calcification inside the fibrous capsule (× 10 hematoxylin and eosin stain; white arrows indicate calcifications). **c** Image around another area of the capsule showing viable cancer cells beneath the capsule (× 10 hematoxylin and eosin stain). **d** Image around the tumor septum showing calcification and viable cancer cells (× 10 hematoxylin and eosin stain; white arrows indicate calcifications)

The resected specimen presented as a solid white tumor with a thick capsule containing rim calcification and necrotic tissue macroscopically (Fig. 2a–c). Histological findings confirmed that necrotic and hemorrhagic tissues were observed in most parts of the tumor. A few viable cancer cells with moderate differentiation were also detected (Fig. 3a–d). The tumor capsule had a thick fibrosis along with rim calcification (Fig. 3b). The patient was discharged uneventfully from our hospital on postoperative day 10 and has survived for 9 years without recurrence.

## Discussion

To our knowledge, ring calcification in hepatocellular carcinoma is rare. There are no more than five previous case reports in the English literature [10–13]. We present the sixth case of primary HCC with ring calcification and review previously published reports with the aim of consolidating the findings relating to its clinical and pathological features (Table 1). Five out of six reported cases were from Japan and derived from infection by the hepatitis C virus. Most of the tumors were no larger than 40 mm in diameter, and ring calcification of the tumors was easily recognized on CT. Surgery was performed in four of the six cases, and survival after surgery presented satisfactory results. Usually, making a correct preoperative diagnosis based on dynamic CT findings, history of viral infection, and patient information is not difficult. Preoperative biopsy should be performed carefully because dissemination after needle biopsy was reported in cases of HCC and echinococcosis [10, 14]. For small HCCs, radio frequency ablation could have been carried out, but radio frequency ablation is unlikely to be the best therapy because of the physical difficulty in targeting an eggshell-like lesion. Thus, radical resection seems to be the best therapy for both ring-calcified HCC and hydatid disease.

**Table 1:** Summary of reported ring-calcified HCC

	Case	Year	Nationality	Age	Sex	Liver condition	Viral infection	Size (mm)	Location	CT pattern	Histology	Treatment	Survival
1	Mitchell et al. [10]	1994	Greek	56	M	LC	HBV	NA	S8	Early enhancement	NA	Chemotherapy	NA
2	Fukuya et al. [11]	1999	Japanese	72	F	CH	HCV	30	S8	Delayed enhancement	Moderately differentiated HCC	Partial hepatectomy	NA
3	Fukuya et al. [11]	1999	Japanese	77	F	LC	HCV	30	S8	Early enhancement	Not obtained	None	NA
4	Kawada [12]	2008	Japanese	67	F	CH	HCV	37	S4	Early enhancement	Moderately differentiated HCC	Left lobectomy	3 years
5	Murakami et al. [13]	2013	Japanese	68	F	CH	HCV	10	S4	No enhancement	Poorly and moderately differentiated HCC	Left medial sectionectomy	30 months
6	Our case	2020	Japanese	60	M	LC	HCV	19	S6	Early enhancement	Moderately differentiated HCC	Partial hepatectomy	9 years

*CT* computed tomography, *M* male, *F* female, *LC* liver cirrhosis, *CH* chronic hepatitis, *HBV* hepatitis B virus, *HCV* hepatitis C virus, *NA* not available, *HCC* hepatocellular carcinoma

Hydatid disease, or echinococcosis, is a zoonosis that is caused mainly by *Echinococcus granulosus* and *Echinococcus multilocularis*. To make a correct diagnosis of echinococcosis, a patient’s social history is an important factor. For example, red foxes, the definitive hosts of *E. multilocularis*, inhabit the northern island of Japan, and more than 95% of echinococcosis patients in Japan are from the Hokkaido area [15]. Serum antibody tests and image findings are also helpful for diagnosis. The sensitivity and specificity of serum antibody tests were reported as 61–97.1% and 61.7–100%, respectively, [16–20]. CT can confirm a hepatic mass and the form of calcification, ring calcification; rim calcification is a characteristic finding of echinococcosis [6, 8, 9]. Magnetic resonance imaging helps distinguish cystic components and solid components; the typical findings of alveolar echinococcosis are multiple small round cysts and solid components with slight enhancement after contrast material injection [21]. In the present case, we made a preoperative diagnosis of echinococcosis based on the patient’s social history and image findings.

Calcification of tumors has been reported and discussed for a long time. Collagen fiber has been considered as one of the factors responsible for tumor calcification [22]. In a recent study of papillary thyroid carcinoma, which often presents with tumor calcification, it was reported that osteopontin-a triggered higher matrix calcification and collagen synthesis [23]. It is well-known that rim capsulation is found in some HCC [24, 25]. The encapsulation around HCC is reported to be composed of type I and type III collagen [26]. The correlation between tumor necrosis and collagen synthesis has also been reported. Ishizaki et al. [27] investigated capsulated HCC and reported expression of procollagen alpha 1 and alpha 3 genes in the capsule and necrotic area of the tumor, which were not observed in non-capsulated HCC. Although the detailed mechanism of calcifications remains uncertain, these studies imply that necrosis and collagen synthesis may be responsible for tumor calcification and encapsulation. We speculate that subsequent calcifications may occur in small parts of capsulated HCC where collagen fibers are rich on its rim, and these will be recognized as ring calcification.

## Conclusion

We report an extremely rare ring-calcified HCC case that mimicked hydatid disease. This case serves as a good reminder of the variety of imaging presentations of HCC.

## Data Availability

All data generated or analyzed during this study are included in this published article.

## Abbreviations

HCC: Hepatocellular carcinoma
CT: Computed tomography
